# Alternative Foods in Cardio-Healthy Dietary Models that Improve Postprandial Lipemia and Insulinemia in Obese People

**DOI:** 10.3390/nu13072225

**Published:** 2021-06-29

**Authors:** Diana Maria Muñoz-Perez, Clara Helena Gonzalez-Correa, Elcy Yaned Astudillo-Muñoz, Gloria Liliana Porras-Hurtado, Maite Sanchez-Giraldo, Jose Lopez-Miranda, Antonio Camargo, Oriol Alberto Rangel-Zuñiga

**Affiliations:** 1 Grupo de Investigación Nutrición, Metabolismo y Seguridad Alimentaria, Departamento de Ciencias básicas de Salud, Universidad de Caldas, Manizales 170004, Colombia; diana.2291424565@ucaldas.edu.co (D.M.M.-P.); clara.gonzalez@ucaldas.edu.co (C.H.G.-C.); 2 Grupo de Investigación NutriOma, Facultad de Ciencias de la Salud, Universidad Libre Pereira, Pereira 660001, Colombia; 3 Grupo de Investigación Gerencia del Cuidado, Facultad de Ciencias de la Salud, Universidad Libre Pereira, Pereira 660001, Colombia; elcyy.astudillom@unilibre.edu.co; 4 Clínica Comfamiliar Risaralda, Pereira 660001, Colombia; gporras@comfamiliar.com; 5 Lipids and Atherosclerosis Unit, Internal Medicine Unit, Reina Sofia University Hospital, 14004 Córdoba, Spain; t32sagim@uco.es (M.S.-G.); md1lomij@uco.es (J.L.-M.); 6 Department of Medicine (Medicine, Dermatology and Otorhinolaryngology), University of Córdoba, 14071 Córdoba, Spain; 7 Maimonides Biomedical Research Institute of Cordoba (IMIBIC), 14004 Cordoba, Spain; 8 CIBER Fisiopatología de la Obesidad y Nutrición (CIBEROBN), Instituto de Salud Carlos III, 28029 Madrid, Spain

**Keywords:** obesity, postprandial lipemia, postprandial insulinemia, avocado, trout, alternative foods, healthy nutrients

## Abstract

Obesity is one of the major health problems worldwide. Following healthy dietary patterns can be difficult in some countries due to the lack of availability of certain foods; thus, alternative foods are needed. Our aim was to evaluate the effect of a dietary pattern consisting of fruit, avocado, whole grains, and trout (FAWGT) on postprandial insulinemia and lipemia in obese Colombian subjects. A randomized controlled crossover study was conducted, in which 44 subjects with BMI ≥ 30 kg/m^2^ followed either a FAWGT diet or a diet high in saturated fat and rich in processed carbohydrates. Levels of lipids and carbohydrates were measured during the postprandial state. The FAWGT diet reduced fasting insulin, VLDL, and HOMA-IR after 8 weeks (*p* < 0.05), while there was a lower postprandial increase in TG, VLDL, and insulin levels after both acute and chronic intake of FAWGT diet (*p* < 0.05). The intake of FAWGT-diet was characterized by high consumption of foods rich in fiber, MUFAs, and vitamins C and E (*p* < 0.05). The consumption of a diet composed of fruit, avocado, whole grains, and trout has emerged as a valid alternative to the foods included in other heart-healthy diets since it improves postprandial lipemia and insulinemia in obese people and has similar beneficial effects to these healthy models.

## 1. Introduction

Overweight and obesity constitute a major health problem in the world [1]. In fact, the World Health Organization (WHO) declared obesity a pandemic after it was reported in 2016 that 39% of the adult population over 18 years old were overweight and 13% were obese [2]. This increased interest in the higher incidence of obesity has arisen because it is the main cause of non-communicable diseases such as type 2 diabetes mellitus, cardiovascular diseases, and some types of cancer, which in turn increase the mortality rate due to these diseases and generate a heavy economic burden on health systems worldwide [3,4]. Obesity involves a complex interaction between genetic and environmental factors, including diet [5,6,7]. The adoption of unhealthy lifestyles and the adherence to a typical Western dietary model, including a low level of physical activity, and the consumption of saturated fats, carbohydrates, and sugary beverages, constitutes the main cause of obesity [8].

In addition, one of the main conditions that influences the development of obesity is the postprandial state, the period from the intake of food to its metabolism, in which there is a continuous fluctuation in the degree of lipemia and glycemia. There is a rapid remodeling of the lipoproteins and a greater number of metabolic adaptations compared to the fasting state [9]. The problem arises because in modern life, people often consume foods more than three times a day, so they remain in the postprandial state for most of the day [5,10], without the opportunity to achieve lipid clearance or glucose homeostasis.

In southern European countries on the Mediterranean coast, a healthy dietary model based on the consumption of olive oil, nuts, vegetables, and fish has been followed for thousands of years. Interventions with the Mediterranean Diet (Med diet) have shown a decrease in triglyceride-rich lipoproteins (TRL) in the postprandial state, with higher triglyceridemia after consumption of a meal rich in monounsaturated fatty acids (MUFA). However, this dietary model is characterized by a rapid clearing of triglyceride levels and a decrease in total cholesterol, LDL cholesterol, and TC/HDL-c ratio in the fasting state [10,11]. Furthermore, studies in Nordic countries show that a healthy Nordic diet lowers LDL cholesterol levels and blood pressure, among other healthy diets [12]. Thus, the beneficial effects of both Mediterranean and Nordic diets have been widely demonstrated [12,13,14]. However, the adoption of these healthy dietary models entails great difficulty in many countries of the world, mainly due to the unavailability or high cost of important foods such as olive oil, red wine, or salmon, among others. This suggests the need to find alternative models that include foods that are local, accessible, and economical in each region and have similar beneficial effects to those observed with other healthy models.

In the Colombian coffee region (Risaralda, Quindío, and Caldas departments), there is a wide variety of fruit and vegetables with cardio-protective potential due to their anti-inflammatory and antioxidant properties [15]. In fact, previous studies have shown a decrease in IL6, CRP, and leptin levels in obese women after 8 weeks of the intake of a diet high in monounsaturated fatty acids and rich in fruit and vegetables typical of the Colombian coffee region [15].

Based on the above, our aim was to evaluate the effect of the consumption of alternative foods such as fruit, avocado, whole grains, and trout, which contain healthy nutrients similar to the specific original foods from Med and Nordic diets, as alternative foods to those included in other healthy models. Additionally, we evaluated the effect of a dietary pattern consisting of fruit, avocado, whole grains, and trout on postprandial insulinemia and lipemia in obese Colombians.

## 2. Materials and Methods

### 2.1. Subjects

The present study included obese subjects between 45 and 60 years old, with a body mass index (BMI) ≥ 30 kg/m^2^, without diagnosis of kidney, liver, thyroid, or diagnosed cardiovascular disease but including hypertensive and dyslipidemic patients. Non-smokers, non-regular alcohol consumers, and patients not participating in weight reduction programs were also included. The primary endpoint was the postprandial triglyceride response. Based on previous studies, 35 individuals had to be included to detect a 15% difference in triglyceride response between the two interventions with 0.05 significance level and 80% power (type II error Z 0.2), assuming a 10% drop-out rate [16]. The recruitment of participants was performed between October 2017 and June 2018, with 1185 clinical records of patients diagnosed with obesity evaluated. After recruitment, 51 people were included in the study, 7 of whom had hypertension and 23 some kind of dyslipidemia. During the dietary intervention, 7 subjects dropped out for personal reasons unrelated to the study, while 44 subjects finished the study (Supplementary Figure S1). The study was carried out at the “Clínica Comfamiliar” located in Pereira, Colombia. All the participants signed an informed consent form before starting the study and were advised to continue their usual physical activity and lifestyle habits. The study protocol was approved by the Ethics Committee of the University of Caldas (University of Caldas registration number: 0406716) and the Clínica Comfamiliar Pereira, in accordance with the Declaration of Helsinki and is registered in ClinicalTrials.gov (NTC04920409).

### 2.2. Study Design

**Figure 1:**
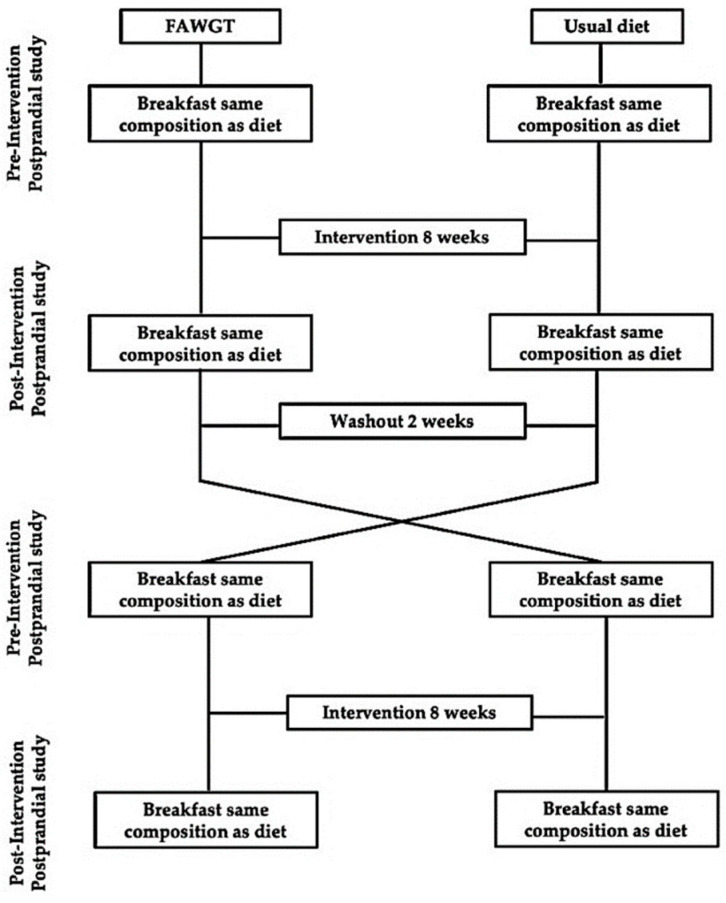
Study design. FAWGT, diet consisting of fruit, avocado, whole grains, and trout.

A randomized controlled crossover study was conducted. Volunteers followed two dietary models for 8 weeks each, including a 2-week washout diet between them. The order in which they started the interventions was randomized following a computerized assignment list using Excel software (Microsoft Office 2015, Excel 2013) (Figure 1). The diets followed during the intervention periods were: (1) a diet consisting mainly of the consumption of fruit, avocado and other vegetables, whole grains, and trout typical of the Colombian coffee region (FAWGT) (experimental diet); and (2) the usual diet consumed by the participants in their normal lifestyle (UD).

### 2.3. Diet Composition

#### 2.3.1. Usual Diet (UD) 

The usual diet consisted of 16% protein, 54% carbohydrates (CH), and 30% fat, of which 15% was saturated fat (SFA), 10% monounsaturated fat (MUFA), and 5% polyunsaturated fat (PUFA) in relation to the total caloric content (TCC). The usual diet was based on the food that the participants usually consumed prior to the study in their normal lifestyle. The determination of the composition of the usual diet was carried out through three 24-h recalls (2 non-consecutive weekdays and one weekend day) before beginning the study.

#### 2.3.2. Washout Diet

The washout diet consisted of returning to the usual diet, characterized by a low consumption of fruit and vegetables <or equal to 2 servings per day, and no consumption of fish or whole grains. The macronutrient distribution remained the same.

#### 2.3.3. Diet Composed of Fruit, Avocado, Whole Grains, and Trout (FAWGT)

The FAWGT diet was composed of 15% protein, 55% CH, and 30% fat, of which <10% was SFA, 14% MUFA, and 6% PUFA in the overall TCC. The FAWGT diet was designed based on foods with antioxidant and anti-inflammatory properties available in the Colombian coffee region, such as fruit and vegetables, trout, and whole grains. The macronutrient content corresponded with the Recommended Energy and Nutrient Intakes (RENIs) for the Colombian population; proteins 14–20%, total fat 20–35%, and carbohydrates 50–65% [17].

In the FAWGT diet, special emphasis was placed on the consumption of fruit and vegetables, legumes, whole grains, canola oil, and fish. To ensure adherence, some food available in the region was provided, including fish (trout), wholegrain cereals (traditionally wholegrain *arepas*—cornmeal pancakes), fats (avocado), and typical fruit from that region (e.g., *granadilla*, *chontaduro*, *uchuvas*, or *carambolo*). In the usual diet, the participants were not given any food but were instructed to eat the foods consumed in their usual diet in their normal lifestyle. The main difference between the diets was the quantity of dietary fiber, and the quality of the fat, carbohydrates, and proteins. A tolerable loss or gain of 1.5 kg of weight was estimated during the study. The food composition of the diets is summarized in Supplementary Table S1.

At the beginning of the dietary intervention period, each participant was given an individualized food guide containing the suggested food group and portions, with a wide variety of foods allowed for greater adherence to the diet. In addition, specific times for consumption of the foods and precise instructions for the dietary intervention were given. In addition, the subjects were given a talk advising them how to quantify the portions, which would later be converted into grams according to the procedure described by Astudillo et al., 2019 [15]. The food portions were standardized with all the participants using synthetic models, adapted according to the Colombian nutritional guidelines [18], so that they could provide a more accurate report of the portions consumed during the intervention.

The Nutritionist Pro software version 7.4.0 (Axxya Systems, Woodinville, WA, USA) was used to calculate energy, macro, and micronutrients. This software includes world food information (USA, Europe, Asia, Central America), and when certain types of Colombian food were not included in the software they were added based on the values provided by Colombian nutritional guides [18].

### 2.4. Postprandial Study

An analysis was carried out at the level of the acute postprandial response, which is defined as the effect from breakfast until 4 h later without a previous period of dietary intervention. A further analysis was carried out of the chronic postprandial response, which is defined as the effect from breakfast until 4 h later after a dietary intervention period of 8 weeks.

The postprandial studies were carried out at the beginning of the study (pre-intervention) and then after 8 weeks of dietary intervention (post-intervention) (Figure 1). The participants were given an appointment at the health center at 7.00 am, after at least 12 h of fasting and a 5-day abstinence from alcohol. They consumed a breakfast based on the same composition of the diet in which they were randomized for the dietary intervention period. The composition of the breakfasts for the postprandial study is shown in Supplementary Table S2. The blood samples were obtained by venipuncture at baseline and 4 h after breakfast. During the postprandial period, the participants did not consume any more food, although they were allowed to drink water. The breakfasts were composed of the following foods. FAWGT diet: whole-grain *arepa* (with unrefined corn flour), cheese, oats, granadilla, mango, linseed, nuts, almonds, peanuts, and yogurt. Usual diet: egg, cheese, butter, whole milk, traditional white *arepa* (with refined corn flour), traditional *buñuelo* (made from wheat flour with cheese), coffee, and sugar.

### 2.5. Nutritional Follow-Up of the Dietary Intervention

Before starting the dietary intervention period (pre-intervention), at the midpoint of the study (week 4) and at the end (week 8), all the participants completed three 24-h recalls (2 non-consecutive weekdays and one weekend day) to obtain information about food, ingredients, and preparations consumed in standard units of measurement (grams). In addition, a weekly telephone call was made to answer any questions relating to the diet (recipes, menu and quantities) and to motivate adherence to the assigned dietary model. Moreover, in week four of each intervention, the participants attended the hospital for an interview with the main researcher in order to take anthropometric measurements, evaluate the follow-up of the dietary instructions, and answer any questions that may have arisen during the intervention, as well as motivating them to continue with the study. To collect the information on food consumption, formats and questionnaires previously published by the research group were used [15]. The protocol to establish adherence to the diet was the three 24-h recalls reminder food record (2 non-consecutive weekdays and one weekend day), which was carried out 5 times during each intervention period: weeks 4, 8, 10, 14, and 18.

### 2.6. Biochemical Measurements of Metabolic Parameters

Venous blood from the participants was collected in tubes containing EDTA after a 12-h overnight fast, and these were placed in containers with ice and kept in the dark. Samples were collected at baseline and 4 h after breakfast, both in the pre-intervention stage and in the post-intervention stages. Immediately after the blood extraction, the plasma was separated by centrifugation at 1500× *g* for 15 min at 4 °C. The plasma samples were aliquoted and stored at −80 °C until the measurements were made, to avoid inter-assay variations.

Lipid variables were assessed using a COBAS Hitachi autoanalyzer using specific reagents (Roche, Basel, Switzerland). The levels of total cholesterol (TC) and triglycerides were measured by colorimetric enzymatic methods, high-density lipoprotein (HDL-c) was measured by colorimetric assay, and low-density lipoprotein (LDL-c) concentration was calculated by the Friedewald equation, using the following formula: LDL-c = TC − (HDL-c + TG/5) [19]. VLDL particles were calculated by the following formula: TG/5. Glucose measurements were performed by the hexokinase method using Roche Diagnostics reagents. Plasma insulin concentrations were measured on a Roche COBAS 6000 system (Roche, Basel, Switzerland). The Homeostatic Model Assessment for Insulin Resistance (HOMA-IR) index was calculated was calculated using the following formula HOMA-IR = Fasting insulinemia (µU/mL) * Fasting glycemia (mg/dL)/405 [20].

### 2.7. Statistical Analysis

All the data are expressed as mean values and standard error. SPSS 20 for Windows (SPSS Inc., Chicago, IL, USA) was used for the statistical analysis. The differences in the baseline characteristics of subjects included in the study were evaluated by a ONE-WAY ANOVA test. The differences between the baseline and the post-intervention period were analyzed using analysis of variance (ANOVA) for repeated measures. The Greenhouse-Geisser contrast statistic was used when the sphericity assumption was not satisfied. In this analysis, we studied the overall diet influence (global ANOVA, *p* for diet influence), the kinetics of the postprandial response (*p* for time), and the interaction of the two factors (diet vs. time). When post hoc test analyses were pertinent, we used multiple comparison tests with the Bonferroni correction. *p* < 0.05 was considered as statistically significant.

## 3. Results

### 3.1. Baseline Characteristics of Subjects Included in the Study

Table 1 shows the anthropometric and biochemical characteristics of the study participants before any dietary intervention at baseline. In addition, Table 2 shows the characteristics of the participants before starting each dietary intervention after the wash-out period. We observed no significant differences in the clinical and anthropometric characteristics, except for levels of HDL cholesterol, which were lower in the group of patients assigned to the FAWGT diet compared with the patients assigned to the usual diet (*p* < 0.001) (Table 2).

### 3.2. Energy Consumption and Dietary Composition after the Intervention Periods

The analysis of energy consumption and dietary composition after 8 weeks with the both interventions (FAWGT and UD) showed an increase in the intake of fiber, MUFA, Omega-3 FA, beta-carotenes, vitamin C, and vitamin E by the consumption of FAWGT diet compared with the UD. In contrast, we observed a lower intake of SFA, Omega-6/Omega-3 ratio, cholesterol, Zn, and Se after 8 weeks of intervention with the FAWGT diet than the UD, all *p* < 0.05 (Table 3).

### 3.3. Acute Postprandial Effect of the Dietary Intervention

**Figure 2:**
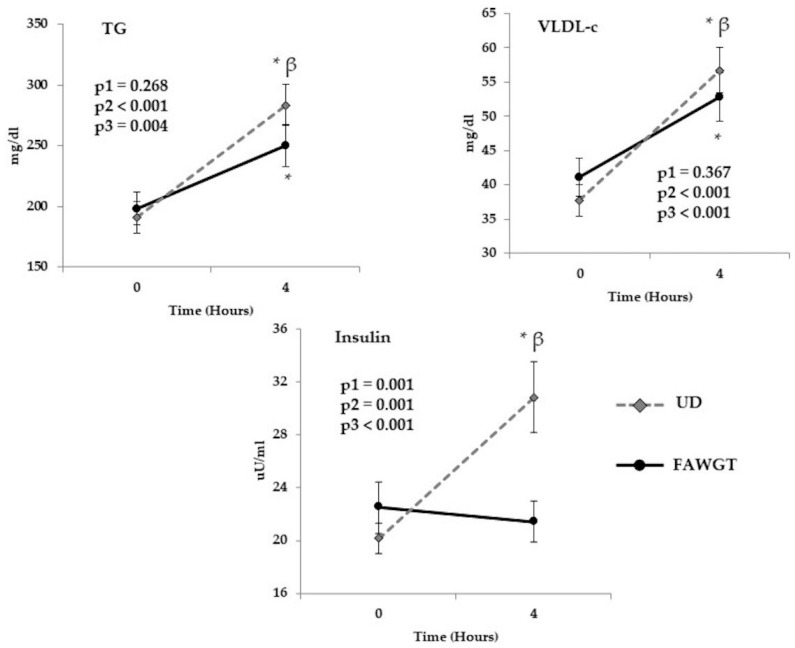
Acute postprandial effect of dietary intervention. Results correspond to the postprandial study performed on the first day of each dietary period. Values are shown as mean ± S.E.M, and the analysis corresponds to an ANOVA of repeated measurements, where p1 = diet influence; p2 = time, kinetics after 4 h; and p3 = the interaction of the two factors (diet vs. time). * *p* < 0.05 = 4 h after breakfast compared to fasting. β = *p* < 0.05 FAWGT vs. UD. FAWGT: diet based on fruit, avocado, whole grains, and trout; UD: usual diet.; TG: triglycerides; VLDL-c; very low-density lipoproteins.

The analysis of the acute postprandial effects of the intake of a diet rich in fruit, avocado, whole grains, and trout available in the Colombian coffee region (FAWGT) compared with a UD was carried out at the beginning of the study (Figure 1) and showed that the two models induced a postprandial increase in triglyceride and VLDL levels after 4 h compared to the fasting state (both diets *p* < 0.001). However, this postprandial increase was lower in both TG and VLDL levels (*p* = 0.046 and *p* = 0.023, respectively) after the acute intake of the FAWGT diet compared to the UD. Moreover, the acute intake of the UD induced a postprandial increase in insulin levels compared to the fasting state (*p* < 0.001). In fact, insulin levels were significantly higher at 4 h after the acute intake of the UD compared with the acute intake of the FAWGT diet (*p* < 0.001) (Figure 2). No significant differences between diets were observed in the other variables analyzed (glucose, total cholesterol, non-HDL-c, LDL-c, and CRP), except for the HDL-c plasma levels, which decreased after the acute consumption of both diets (Figure S2).

### 3.4. Chronical Postprandial Effect of Dietary Intervention

**Figure 3:**
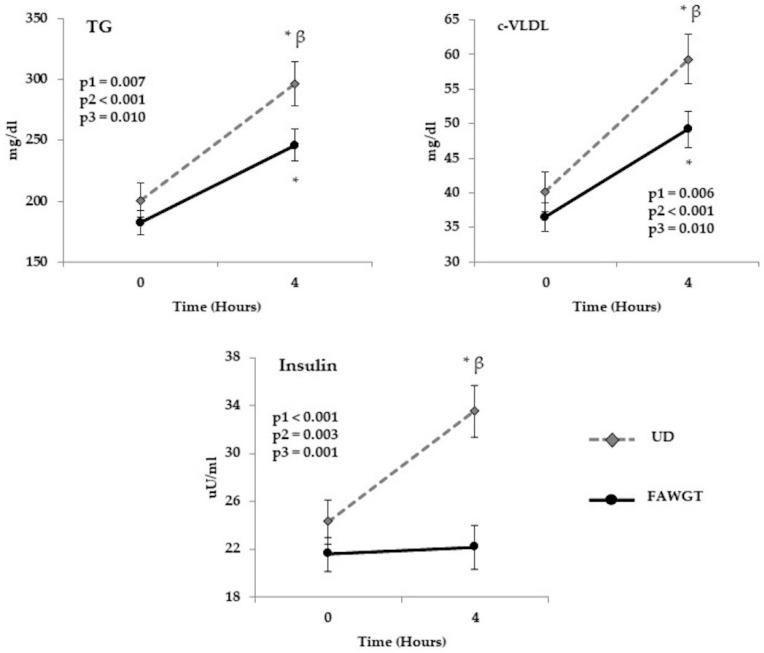
Chronic postprandial effect of dietary intervention. Results correspond to the postprandial study performed on the last day of each dietary period. Values are shown as mean ± S.E.M, and the analysis corresponds to an ANOVA of repeated measurements, where p1 = diet influence; p2 = time, kinetics after 4 h; and p3 = the interaction of the two factors (diet vs. time). * *p* < 0.05 = 4 h after breakfast compared to fasting. β = *p* < 0.05 FAWGT vs. UD. FAWGT: diet based on fruit, avocado, whole grains, and trout; UD: usual diet.; TG: triglycerides; VLDL-c; very low-density lipoproteins.

Further, we analyzed at the postprandial state the chronic effect of the consumption for 8 weeks of the FAWGT or the usual diet (chronic postprandial effect), which showed an increase at 4 h with respect to baseline in the levels of triglyceride and c-VLDL after the intake of the two diets (both *p* < 0.001). However, this increase was lower after the intake of the FAWGT diet than the UD, in both triglyceride and VLDL levels (both *p* = 0.001). Finally, after 8 weeks of dietary intervention, the usual diet induced an increase in insulin levels at 4 h after breakfast compared with baseline (*p* = 0.002). This increase was statistically significant between both diets at 4 h of the postprandial state (*p* < 0.001) (Figure 3). No significant differences between diets were observed in the other variables analyzed (blood pressure, glucose, total cholesterol, HDL-c, non-HDL-c, and CRP), except for the LDL-c plasma levels, which were higher in FAWGT diet (Figure S3).

### 3.5. Chronic Effect of Dietary Intervention after 8 Weeks

**Figure 4:**
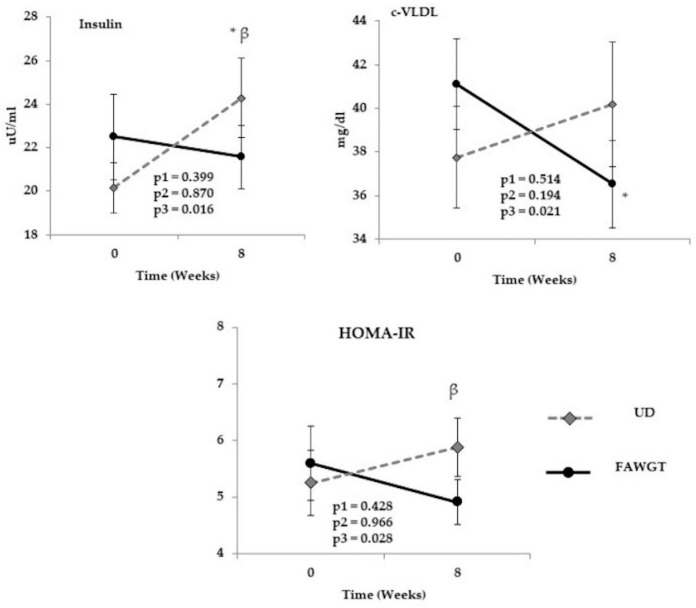
Chronic effect of the intake of a diet based on fruit, avocado, whole grains, and trout after 8 weeks of intervention. Values are shown as mean ± S.E.M, and the analysis corresponds to an ANOVA of repeated measurements, where p1 = diet influence; p2 = time, kinetics after the intervention period in fasting state; and p3 = the interaction of the two factors (diet vs. time). * *p* < 0.05 after 8 weeks of intervention compared to baseline. β = *p* < 0.05 FAWGT vs. UD. HOMA-IR: Homeostatic Model Assessment for Insulin Resistance; FAWGT: diet based on fruit, avocado, whole grains, and trout; UD: Usual diet.

The analysis of the fasting status after 8 weeks of intervention showed that the usual diet (UD) increased the insulin levels after the intervention period compared to the baseline (*p* = 0.006). This difference was statistically significant compared with the FAWGT diet after 8 weeks of intervention (*p* = 0.018). Additionally, the FAWGT diet induced a decrease in VLDL levels after the intervention period compared to the baseline (*p* = 0.026). Insulin resistance, assessed by the HOMA-IR index, was lower after 8 weeks of the FAWGT diet than the UD (*p* = 0.013) (Figure 4). Moreover, we found a reduction on body weight after the FAWGT diet (*p* < 0.001), in parallel with decrease in BMI (*p* < 0.001), whereas we observed an increase in BMI after the UD period (*p* < 0.001). We also observed a decrease in HDL-c levels after the UD period (*p* = 0.039). No statistically significant differences were found in fat percentage, waist hip index, blood pressure, glucose, total cholesterol, non-c HDL-c, LDL-c, triglycerides, and C-reactive protein (Table S3).

## 4. Discussion

Our study shows that the intake of a diet composed by fruit, avocado, whole grains, and trout could emerge as a valid alternative to other healthy dietary patterns, as it led to an improvement in postprandial lipemia and insulinemia and a decrease in insulin resistance in obese people but without causing clinically significant weight loss. This was supported by the fact that consumption of an FAWGT diet increased the quantity of healthy fatty acids such as MUFA, Omega 3, and fiber, as well the quantity of molecules with antioxidant power, such as vitamins C and E. Additionally, the FAWGT diet reduced the increase in postprandial triglycerides (TG) and VLDL levels after both acute and chronic intake compared to the usual diet. Additionally, the intake of the FAWGT diet prevented an increase in postprandial insulin levels in both the acute and chronic intervention in contrast to the UD, without significant differences between diets in postprandial glucose increases after both acute and chronic intake. Finally, these results were obtained in free-living conditions with food available in the region, without the use of any food supplements, which would allow us to generalize these findings.

Southern European countries on the coast of the Mediterranean Sea advise their populations to consume the Mediterranean diet, which is rich in extra virgin olive oil, fish, fruit, nuts, vegetables and legumes [21]. Additionally, the Nordic diet recommends the frequent consumption of fruit, berries, vegetables, legumes, potatoes, whole grains, nuts, seeds, rye breads, fish, seafood, low-fat dairy, herbs, spices, and rapeseed (canola) oil [22]. Both of these dietary models help to prevent high blood pressure, lower cholesterol levels and obesity-associated low-grade chronic inflammation, and reduce the risk of cardiovascular disease and type 2 diabetes mellitus. The diets are considered healthy dietary models and are based on common foods in these regions that are not always easily available in other countries. However, there is a need to implement dietary models with alternative foods with a similar nutrient content that have similar metabolic benefits in terms of glucose and lipids.

Previous studies have shown that the Mediterranean diet improves the lipid profile during the postprandial state [14,21]. In fact, it has been suggested that the consumption of the monounsaturated fatty acids (MUFA) present in olive oil decreases postprandial glucose and insulin concentrations after breakfast and increases HDL cholesterol (HDL-c) and glucagon-like pepide-1 (GLP-1) concentrations compared with a carbohydrate-rich diet [23]. GLP-1 decreases lipid and glucose levels by enhancing insulin secretion and synthesis and improves pancreatic β-cell proliferation. Other studies show that this proliferation is progressive as the MUFA to SAFA ratio increases [23,24]. Other studies have shown that participants who ingest 20% of their energy as MUFA have an earlier increase and a faster clearance of postprandial plasma TG and large concentrations of triglyceride-rich lipoproteins (TRL-TG) compared with SFA and low-fat diets [25]. In our study, we observed a reduced increase in TG and VLDL in both acute and chronic postprandial state after the intake of the FAWGT diet model; in addition, there was a decrease in fasting VLDL after 8 weeks of intervention with the same diet. This effect could be attributable to the increase in MUFA intake from 10.8 to 12.1%, whose main source was avocado, with an intake of 18 g/d. These findings are consistent with those found by Anderson-Vasquez, HE et al., where the addition of 250 g avocado to the usual diet decreased the levels of total cholesterol, LDL-c and TG [26]. Similarly, our study showed an increase in omega-3 intake and a decrease in the omega-6/omega-3 ratio from 12:1 to 6:1, possibly due to the consumption of 280 g/week of trout. The replacement of olive oil by avocado and the consumption of trout could have similar effects on lipemia and postprandial insulinemia to those found with established healthy diets, considering that both are an important source of MUFA (55–83% and 63%, respectively).

In addition, one previous study demonstrated a postprandial decrease of insulin and triglycerides after replacing refined grains with whole grains to achieve a fiber consumption of 40 g/d, although this reduction was not parallel to the reduction of glucose [27]. Sun et al. found a decrease in postprandial insulin regardless of the degree of fatty acid saturation after the intake of a combination of 50 g carbohydrates (low and high glycemic index) and 40 g fat (SAFA, MUFA, and PUFA) [28]. In this context, the contribution of our study is to suggest that the consumption of *arepa* with whole-grain corn, brown rice, and oatmeal of 115 g/d increases the fiber intake. This was higher in the FAWGT (32 g/d) than the UD (15 g/d). This effect could be due to fiber being metabolized in the intestinal microbiota, whose products are short-chain fatty acids, especially butyrate and propionate, which could improve insulin sensitivity [29,30]. Moreover, this potentially favorable effect of the FAWGT diet could also reduce the cardiovascular risk by reducing insulin resistance, as recently described [31]. Similarly, this alternative dietary model was able to decrease the acute and chronic postprandial insulin response, which suggests an improved glucose metabolism, as evidenced by the HOMA-IR index. However, further research is needed to ascertain why no significant differences in glucose levels were observed.

Finally, fruit consumption is associated with an increase in antioxidant capacity, increasing the potential for the elimination of reactive oxygen species, which show high levels in obese people. Vitamin C and E decrease oxidative damage, inhibiting lipid peroxidation and maintaining pancreatic β cell function, which may inhibit gluconeogenesis and glycogenolysis [32]. Likewise, vitamin C may increase the action of lipoprotein lipase in adipose tissue and decrease levels of TG, VLDL, and LDL cholesterol [33]. This study showed an increase in vitamin C and E levels from 91.2 to 207.13 mg and from 4.43 to 7.01 mg, respectively (*p* < 0.001). The main sources of these vitamins were the typical fruits of the Colombia coffee region, such as mango, orange, purple passion fruit, and *chontaduro*.

FAWGT diet was based in the consumption of typical arepa, rice, oat, lentils, tomato, carrot, lettuce, onion, beans, avocado, trout, mango, orange, apple, pear, tangerine, pineapple, papaya, *chontaduro*, *uchuva*, *carambolo*, and *granadilla*, which altogether add fiber, antioxidant, vitamins, and MUFA in different proportions. Therefore, it is complex to discern which specific component is driving the differences found between FAWGT and the usual diet as several foods were introduced.

Our study has also other limitations. The sample size was low, although the experimental randomized crossover design allowed us to detect the effect of the intake of a diet composed by fruit, avocado, whole grains, and trout. However, this fact, together with the short duration of each dietary period, may limit the extrapolation of these findings, and therefore further studies would be needed to confirm them.

## 5. Conclusions

In conclusion, the consumption of a diet composed of fruit, avocado, vegetables, whole grains, and trout can be considered a valid alternative to other heart-healthy diets, since it improves postprandial lipemia and insulinemia in obese people without causing clinically significant weight loss and shows similar beneficial effects to these healthy models.

## Figures and Tables

**Table 1:** Baseline characteristics of the patients included in the study before any dietary intervention.

	Total *n* = 44	Women *n* = 34	Men *n* = 10
Age	50.8 ± 6.3	50.5 ± 6.5	51.9 ± 5.5
Weight (kg)	88.6 ± 13.5	87.0 ± 13.7	94.4 ± 11.5
Waist-hip ratio	0.91 ± 0.1	0.89 ± 0.1	0.97 ± 0.1
Body mass index (kg/m^2^)	35.6 ± 4.2	36.2 ± 4.3	33.7 ± 3.3
Fat (%)	42.3 ± 4.2	43.6 ± 2.7	37.9 ± 5.7
Systolic blood pressure (mmHg)	122.7 ± 13.4	121.0 ± 12.3	128.7 ± 16.1
Diastolic blood pressure (mmHg)	81.1 ± 9.0	79.5 ± 8.4	86.6 ± 9.2
Handgrip strength (kg)	26.6 ± 7.7	24.6 ± 5.7	33.7 ± 9.8
Insulin (µU/mL)	20.8 ± 14.0	18.6 ± 7.3	28.15 ± 25.83
Glucose (mg/dL)	95.1 ± 11.1	93.9 ± 9.5	99.1 ± 15.2
HOMA-IR	5.0 ± 3.9	4.4 ± 1.9	7.3 ± 7.2
TC (mg/dL)	203.5 ± 37.4	203.4 ± 38.8	203.9 ± 34.2
HDL-c (mg/dL)	43.1 ± 10.5	44.1 ± 11.2	39.7 ± 6.6
Non- HDL-c (mg/dL)	160.4 ± 38.8	159.2 ± 41.1	164.2 ± 31.0
LDL-c (mg/dL)	122.2 ± 35.4	121.7 ± 36.8	124.1 ± 32.1
VLDL-c (mg/dL)	38.2 ± 18.1	37.6 ± 16.4	40.1 ± 24.0
TG (mg/dL)	190.8 ± 90.4	187.9 ± 81.8	200.4 ± 120.0
hs-CRP (mg/L)	5.1 ± 5.1	5.8 ± 5.6	2.8 ± 1.2

Data shows the mean ± SD. HOMA-IR: Homeostatic Model Assessment for Insulin Resistance; TC: total cholesterol; HDL-c: high-density lipoproteins; LDL-c: low-density lipoproteins; VLDL-c: very low-density lipoproteins; TG: triglycerides; hs-CRP: C-reactive protein.

**Table 2:** Characteristics of the participants before starting each dietary intervention after the wash-out period.

	FAWGT	UD	*p* Value
N	44	44	n.a.
Weight (kg)	88.5 ± 13.5	88.3 ± 13.7	0.322
Waist-hip ratio	0.90 ± 0.1	0.91 ± 0.1	0.128
Body mass index (kg/m^2^)	35.7 ± 4.3	35.5 ± 4.3	0.206
Fat (%)	42.7 ± 3.5	42.4 ± 4.5	0.341
Systolic blood pressure (mmHg)	122.1 ± 11.7	120.1 ± 12.6	0.343
Diastolic blood pressure (mmHg)	78.6 ± 9.3	80.1 ± 8.0	0.224
Handgrip strength (kg)	26.9 ± 7.3	27.7 ± 8.2	0.261
Insulin (µU/mL)	22.5 ± 13.0	20.2 ± 7.8	0.239
Glucose (mg/dL)	94.3 ± 10.9	95.9 ± 11.0	0.221
HOMA-IR	5.6 ± 3.7	5.3 ± 2.3	0.591
TC (mg/dL)	201.3 ± 36.5	202.0 ± 35.3	0.811
HDL-c (mg/dL)	40.1 ± 9.9	43.4 ± 10.7	<0.001
Non- HDL c (mg/dL)	161.0 ± 38.2	159.2 ± 36.4	0.572
LDL-c (mg/dL)	118.4 ± 36.1	118.0 ± 34.3	0.922
VLDL-c (mg/dL)	41.1 ± 18.4	37.7 ± 15.5	0.218
TG (mg/dL)	198.2 ± 88.8	191.2 ± 86.3	0.811
hs-CRP (mg/L)	4.3 ± 2.5	4.6 ± 2.4	0.248

Data shows the mean ± SD. FAWGT: diet composed of fruit, avocado, whole grains, and trout; UD: usual diet. HOMA-IR: Homeostatic Model Assessment for Insulin Resistance; TC: total cholesterol; HDL-c: high-density lipoproteins; LDL-c: low-density lipoproteins; VLDL-c: very low-density lipoproteins; TG: triglycerides; hs-CRP: C-reactive protein.

**Table 3:** Dietary composition at baseline and after 8 weeks of intervention.

	FAWGT		UD				
	0 Week	8 Week	0 Week	8 Week	*p* Diet	*p* Time	*p* Interaction
Energy (Kcal)	1774.2 ± 265.5	1627.9 ± 152.9 (a)	1900.2 ± 216.7	1800.6 ± 204.7 (a) (b)	<0.001	<0.001	0.374
Protein (E%)	16.6 ± 2.3	16.7 ± 2.5	16.5 ± 2.6	17.2 ± 1.7	0.492	0.212	0.337
Carbohydrates (E%)	50.8 ± 5.1	53.6 ± 4.7 (a)	53.7 ± 4.4	53.8 ± 3.9	0.012	0.030	0.054
Total Fiber (g)	13.6 ± 4.1	32.9 ± 5.8 (a)	14.4 ± 4.4	14.6 ± 3.9 (b)	<0.001	<0.001	**<0.001**
Total Fat (E%)	30.6 ± 6.4	29.3 ± 3.8	29.7 ± 3.6	29.0 ± 3.1	0.339	0.072	0.673
SFA (E%)	10.1 ± 2.3	7.4 ± 1.5 (a)	10.8 ± 2.1	10.6 ± 1.6 (b)	<0.001	<0.001	**<0.001**
MUFA (E%)	10.8 ± 2.2	12.1 ± 1.9 (a)	9.2 ± 1.6	9.1 ± 1.5 (b)	<0.001	0.018	**0.011**
PUFA E (%)	6.9 ± 2.5	6.7 ± 1.4	4.9 ± 1.1	4.7 ± 1.1 (b)	<0.001	0.448	0.956
Linoleic acid (g)	11.6 ± 4.4	9.8 ± 1.8 (a)	8.5 ± 2.4	8.1 ± 3.1 (b)	<0.001	0.019	0.166
Alpha-linolenic acid (g)	1.0 ± 0.4	1.6 ± 0.7 (a)	0.9 ± 0.3	0.8 ± 0.3 (b)	<0.001	0.019	**<0.001**
Ω6/Ω3 ratio	11.9 ± 2.8	7.0 ± 3.0 (a)	10.0 ± 1.8	11.0 ± 6.2 (b)	0.062	0.003	**<0.001**
Cholesterol (mg)	302.3 ± 136.2	242.9 ± 83.0 (a)	322.9 ± 139.0	333.3 ± 117.0 (b)	<0.001	0.151	**0.023**
Beta-carotene (µg)	1918.1 ± 1307.6	6436.3 ± 2806.6 (a)	1790.1 ± 1121.4	1788.4 ± 1021.2 (b)	<0.001	<0.001	**<0.01**
Vitamin C (mg)	91.5 ± 66.8	207.1 ± 57.8 (a)	108.7 ± 65.7	92.2 ± 41.4 (b)	<0.001	<0.001	**<0.001**
Vitamin E (mg)	4.4 ± 1.6	7.0 ± 1.7 (a)	3.9 ± 1.1	3.6 ± 0.9 (b)	<0.001	<0.001	**<0.001**
Folate (µg)	270.4 ± 101.8	305.8 ± 69.7 (a)	272.6 ± 90.7	285.2 ± 86.7	0.321	0.037	0.441
Mg (mg)	206.0 ± 48.5	294.4 ± 50.2 (a)	227.3 ± 47.1	220.8 ± 38.3 (b)	0.321	0.037	0.441
Zn (mg)	8.6 ± 2.6	7.2 ± 1.2 (a)	8.1 ± 3.3	8.8 ± 2.5 (b)	0.042	0.340	**0.001**
Se (µg)	77.0 ± 20.4	63.4 ± 13.5 (a)	79.2 ± 22.5	81.7 ± 20.8 (b)	<0.001	0.018	**0.002**

Values expressed as mean ± SD. FAWGT: diet composed of fruit, avocado, whole grains, and trout; UD: usual diet; SFA: saturated fatty acids; MUFA: monounsaturated fatty acids; PUFA: polyunsaturated fatty acids; Mg: magnesium; Zn: zinc; Se: selenium. Variables were calculated using a repeated measurement analysis through SPSS (now PASW Statistic for Windows, version 21.0) (IBM, Chicago, IL, USA). In bold *p* < 0.05 in the interaction diets vs. time. (a) *p* < 0.05 relative to baseline values in the diet. (b) *p* < 0.05 between diets in the same time period.

## Data Availability

The data presented in this study are available on request from the corresponding author and to the principal researchers of the project D.M.M.-P. and C.H.G.-C.

## Supplementary Materials

The following are available online at https://www.mdpi.com/article/10.3390/nu13072225/s1, Figure S1: Flow chart of study participants, Figure S2: Acute postprandial effect of dietary intervention on other biochemical variables, Figure S3: Chronic postprandial effect of dietary intervention on other biochemical variables, Table S1: Recommended foods in the study, Table S2: Composition and caloric distribution of the breakfasts used in the postprandial study, Table S3: Characteristics of subjects included in the study after and before the dietary intervention
