# Supplementary material for: Alternative Foods in Cardio-Healthy Dietary Models that Improve Postprandial Lipemia and Insulinemia in Obese People
Source: Nutrients. 2021 Jun 29;13(7):2225. doi: 10.3390/nu13072225 (PMC8308459; doi:10.3390/nu13072225)
Supplement: Supplementary file 1 [file nutrients-13-02225-s001.zip › FIGURE S1.pdf]

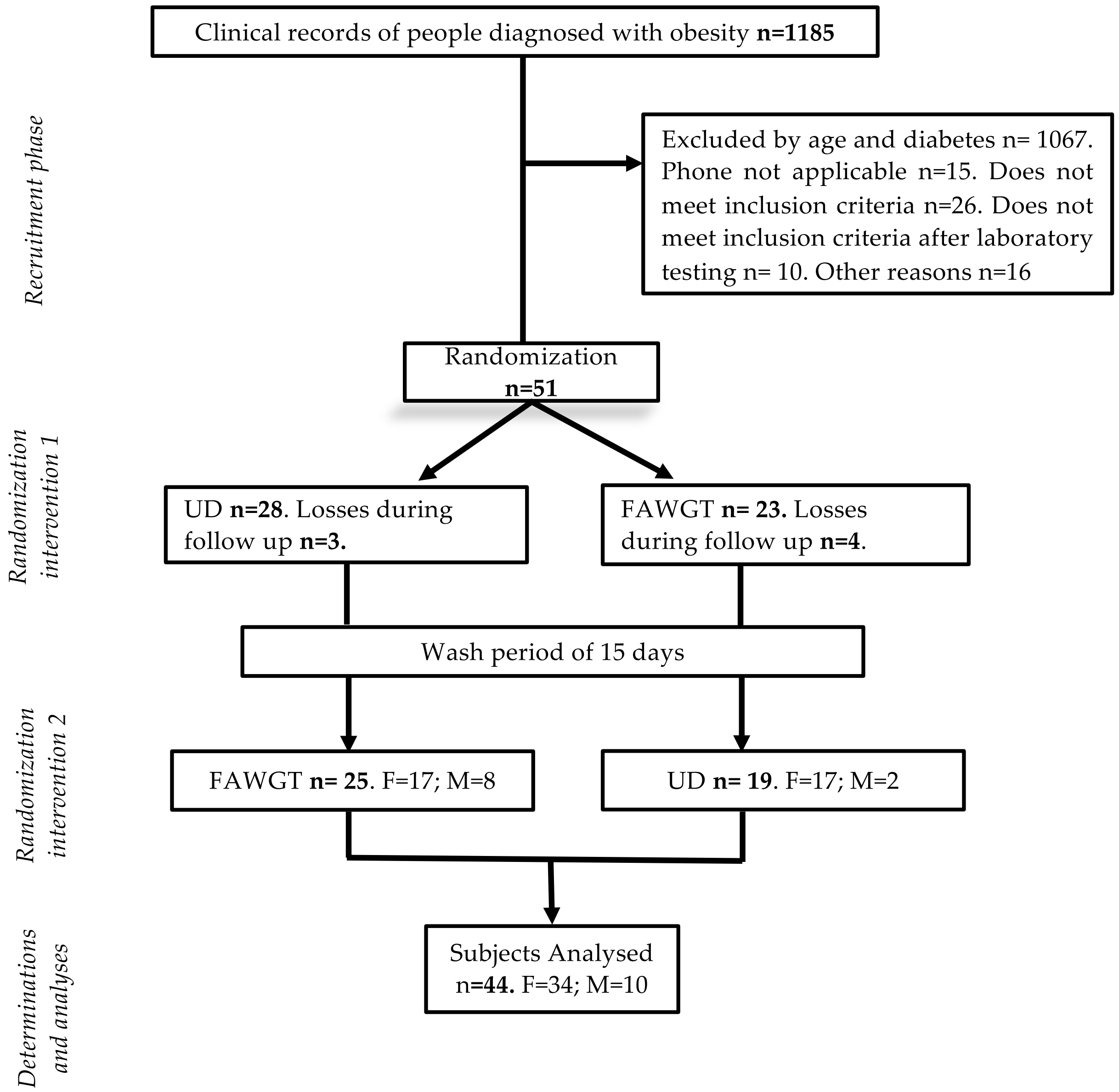

Figure S1. Flow chart of study participants. Source CONSORT 2010. F, Female; M, Males; **UD**, Usual diet; **FAWGT**, Diet composed of fruit, avocado, whole grains and trout.
