# Supplementary material for: Alternative Foods in Cardio-Healthy Dietary Models that Improve Postprandial Lipemia and Insulinemia in Obese People
Source: Nutrients. 2021 Jun 29;13(7):2225. doi: 10.3390/nu13072225 (PMC8308459; doi:10.3390/nu13072225)
Supplement: Supplementary file 1 [file nutrients-13-02225-s001.zip › FIGURE S2.pdf]

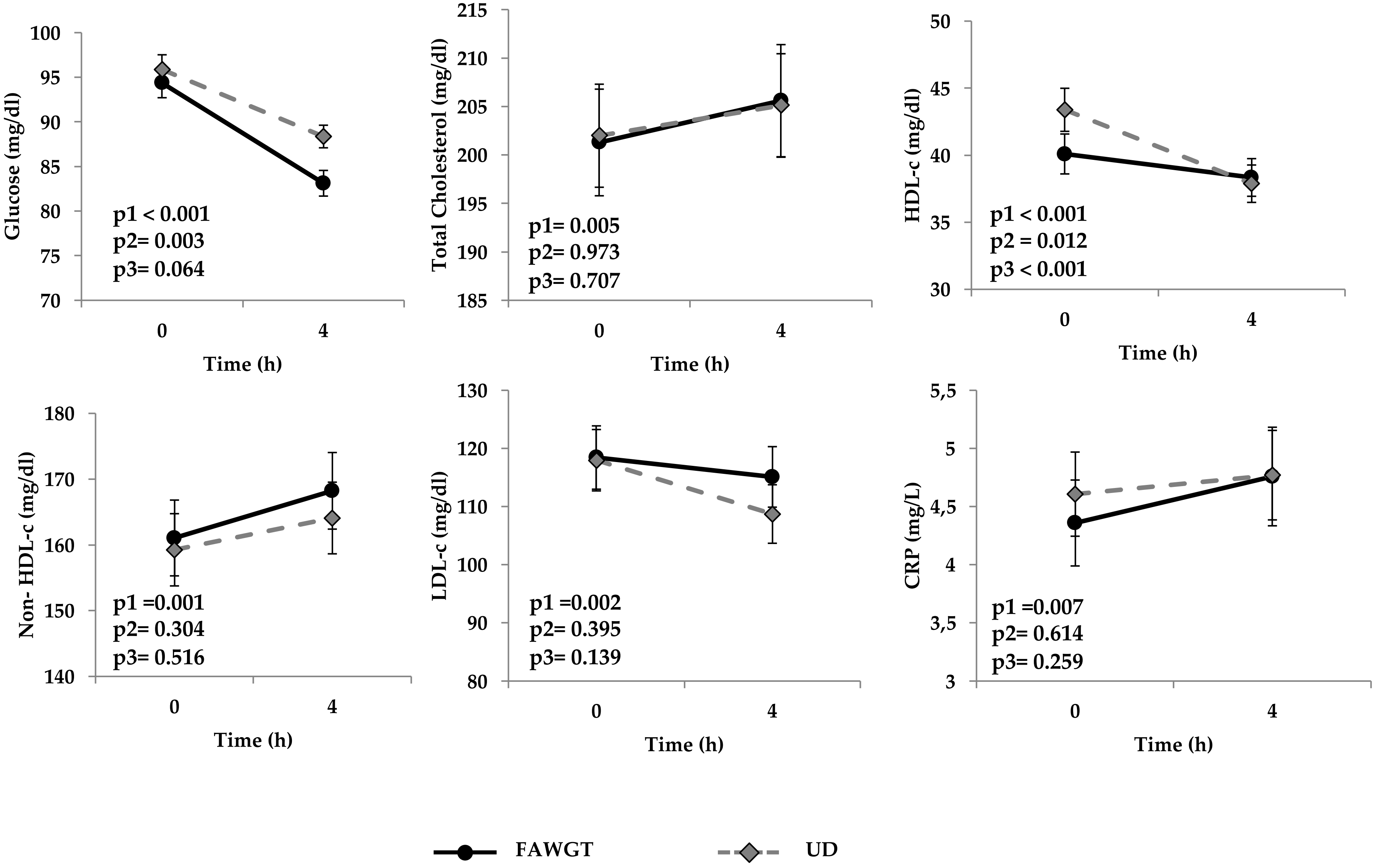


**Figure S2**. Acute postprandial effect of dietary intervention on other biochemical variables. Results correspond to the postprandial study performed the first day of each dietary period. Values are shown as mean ± S.E.M and the analysis corresponds to an ANOVA of repeated measurements, where p1= time, kinetics after 4 hours; p2= diet influence; p3= the interaction of the two factors (diet vs time). FAWGT: Diet based on fruit, avocado, whole grains and trout; UD: Usual diet.; TG:
