# Supplementary material for: Alternative Foods in Cardio-Healthy Dietary Models that Improve Postprandial Lipemia and Insulinemia in Obese People
Source: Nutrients. 2021 Jun 29;13(7):2225. doi: 10.3390/nu13072225 (PMC8308459; doi:10.3390/nu13072225)
Supplement: Supplementary file 1 [file nutrients-13-02225-s001.zip › FIGURE S3.pdf]

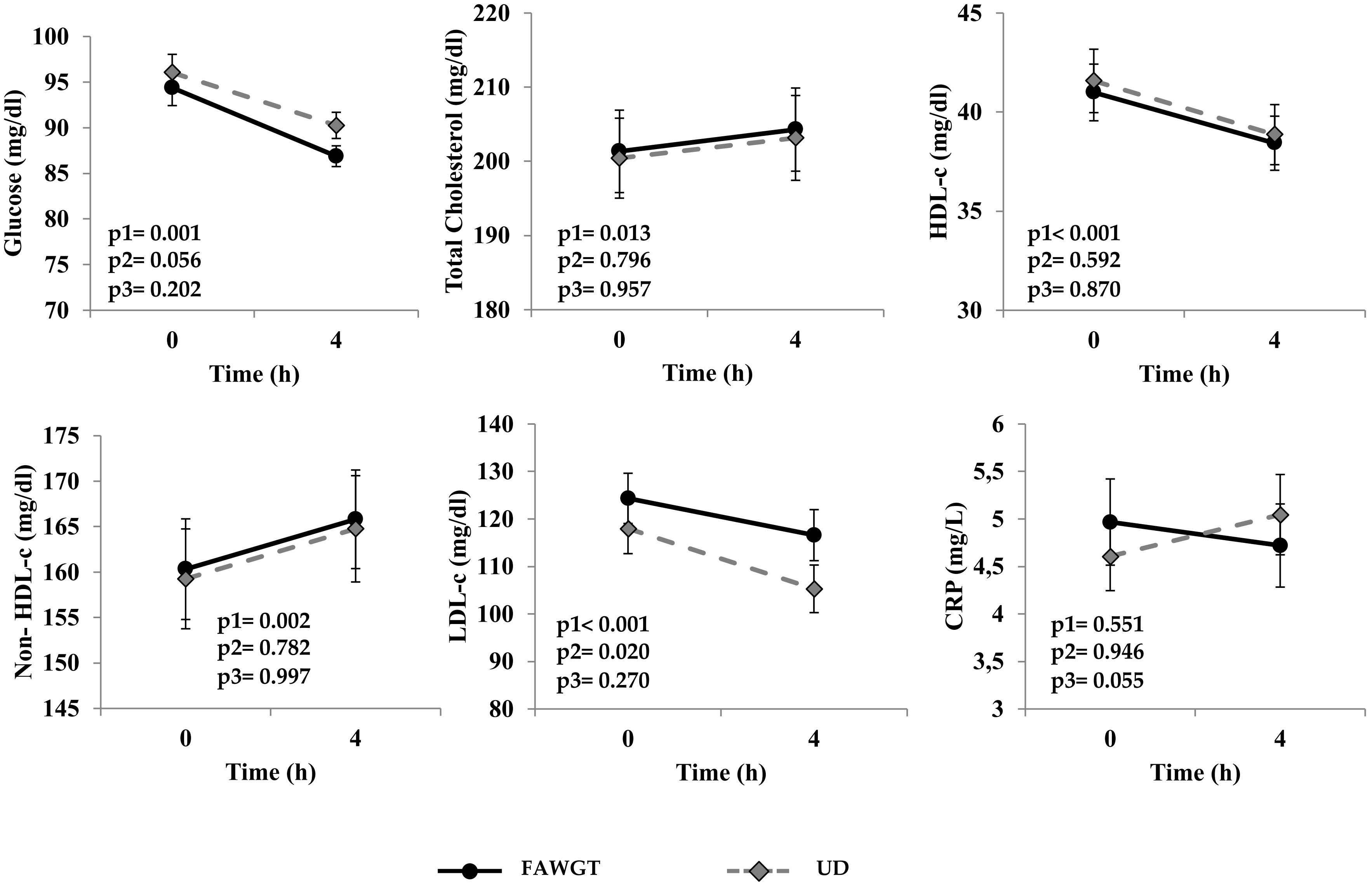

Figure S3. Chronic postprandial effect of dietary intervention on other biochemical variables. Results correspond to the postprandial study performed the first day of each dietary period. Values are shown as mean ± S.E.M and the analysis corresponds to an ANOVA of repeated measurements, where p1= time, kinetics after 4 hours; p2= diet influence; p3= the interaction of the two factors (diet vs time). FAWGT: Diet based on fruit, avocado, whole grains and trout; UD: Usual diet.; TG:
