# Supplementary material for: Alternative Foods in Cardio-Healthy Dietary Models that Improve Postprandial Lipemia and Insulinemia in Obese People
Source: Nutrients. 2021 Jun 29;13(7):2225. doi: 10.3390/nu13072225 (PMC8308459; doi:10.3390/nu13072225)
Supplement: Supplementary file 1 [file nutrients-13-02225-s001.zip › Table S1.pdf]

Table S1. Recommended foods in the study

Food Group	FAWGT	UD
Cereals	Replacement of 50% of refined cereals by whole grains (rice, typical *arepa* and oats): rice of 80 grs, 1 *arepa* of 56 grs and oat of 24 grs per serving	Refined
Fat source	Canola, sunflower or soybean oil 5 grs per serving. Almonds 9 grs, nuts 8 grs and peanuts 10 grs per serving. Avocado 30 grs per serving.	Butter > 50% saturated fat
Fruits and Vegetables	Fruits 400 grs per serving (granadilla, *uchuvas*, orange or tangerine, guayaba, mango, apple) and vegetables 200 grs per serving (citron, spinach, beans, peas, carrots, tomatoes, onions, garlic). Typical *chontaduro* 27 grs, granadilla 100 grs, orange 147 grs per serving. Mango 112 grs, guayaba criolla 105 grs per serving. Carrot 58 grs, tomato 118 grs, beans 81 grs, peas 42 grs, onion 74 grs per serving.	< or equal 200 g/day of fruits and vegetables
Fish	3 portions per week (trout or tuna). Trout of 80 grs per serving or tuna 120 grs per serving.	< 1 portion per week of any type of fish
Meat	Preferably white meat, beef once a week. Meat 100 grs per serving or chicken 80 grs per serving.	On demand, consumption of any type of meat during the week
Legumes	Two times for week (lentils, beans, chickpeas, blanquillos) 120 grs per serving.	< 2 times per week
Beverages	Fruit juice (blackberry, tree tomato, lulo, sweet and sour guayaba) once a day without sugar or sweeteners of 200 cm^3 per serving.	No restrictions

FAWGT: Diet composed of fruit, avocado, whole grains and trout; UD: Usual diet.
