# Supplementary material for: Alternative Foods in Cardio-Healthy Dietary Models that Improve Postprandial Lipemia and Insulinemia in Obese People
Source: Nutrients. 2021 Jun 29;13(7):2225. doi: 10.3390/nu13072225 (PMC8308459; doi:10.3390/nu13072225)
Supplement: Supplementary file 1 [file nutrients-13-02225-s001.zip › Table S2.pdf]

Table S2. Composition and caloric distribution of the breakfasts used in the postprandial study.

		FAWGT	UD
Kilocalories	Kcal	701	717
Proteins	%	12.2	16.7
Carbohydrates	%	47.9	45.5
Fat	%	39.9	37.7
SFA	%	9.6	17.9
MUFA	%	12.96	11.4
PUFA	%	12.71	3.38
Fiber	g	16.3	0.7
β-carotenes	mcg	846	27.6

FAWGT, Diet consisting of fruits, avocado, whole grains and trout; UD: Usual diet; SFA: Saturated fatty acids; MUFA: Monounsaturated fatty acids; PUFA: Polyunsaturated fatty acids.
