# Supplementary material for: Alternative Foods in Cardio-Healthy Dietary Models that Improve Postprandial Lipemia and Insulinemia in Obese People
Source: Nutrients. 2021 Jun 29;13(7):2225. doi: 10.3390/nu13072225 (PMC8308459; doi:10.3390/nu13072225)
Supplement: Supplementary file 1 [file nutrients-13-02225-s001.zip › Table S3.pdf]

	FAWGT		UD				
**Parameter**	**Baseline**	**8 weeks**	**Baseline**	**8 weeks**	**p time**	**p diet**	**p Diet vs Time**
Weight (kg)	88.5 ± 13.5	86.9 ± 13.1 (a)	88.3 ± 13.7	88.4 ± 13.7 (b)	<0.001	0.007	<0.001
BMI (kg/m^2)	35.7 ± 4.3	35.1 ± 4.5 (a)	35.5 ± 4.3	35.6 ± 4.4 (b)	0.001	0.015	<0.001
Fat (%)	42.7 ± 3.5	41.8 ± 3.9	42.4 ± 4.4	42.1 ± 4.0	0.006	0.748	0.285
Waist hip index	0.9 ± 0.1	0.9 ± 0.1	0.9 ± 0.1	0.9 ± 0.1	0.488	0.017	0.764
Sistolic blood (mmHg)	122.1 ± 11.7	119.8 ± 10.6	120.1 ± 12.6	119.9 ± 12.9	0.267	0.539	0.364
Diastolic blood (mmHg)	78.6 ± 9.3	77.6 ± 7.8	80.1 ± 8.0	77.6 ± 8.9	0.850	0.450	0.313
Glucose (mg/dl)	94.3 ± 10.9	94.4 ± 13.0	95.9 ± 11.0	96.0 ± 13.3	0.923	0.079	0.935
Total Cholesterol (mg/dl)	201.3 ± 36.5	201.3 ± 36.9	202.0 ± 35.3	200.4 ± 35.8	0.764	0.962	0.765
HDL-c (mg/dl)	40.1 ± 9.9	41.0 ± 9.5	43.4 ± 10.7 (b)	41.6 ± 10.6 (a)	0.494	0.005	0.039
Non-c HDL-c (mg/dl)	161.1 ± 38.2	160.3 ± 36.7	159.3 ± 36.4	159.0 ± 36.9	0.840	0.525	0.936
LDL-c (mg/dl)	118.4 ± 36.1	124.3 ± 35.0	118.0 ± 35.0	118.8 ± 31.6	0.212	0.303	0.439
TG (mg/dl)	198.2 ± 88.8	182.7 ± 67.2	191.2 ± 86.4	200.9 ± 94.8	0.726	0.444	0.100
CRP (mg/L)	4.4 ± 2.5	5.0 ± 3.0	4.6 ± 2.4	5.2 ± 2.8	0.002	0.326	0.898

Table S3. Characteristics of subjects included in the study after and before the dietary intervention. FAWGT, Diet rich in fruits, avocado, whole grains and trout. UD, Usual diet. Values represent the mean ± standard deviation. The analyses correspond to ANOVA for repeated measures where were investigated p time, kinetics of the dietary intervention response; p diet influence, and p value of the interaction of the two factors (diet vs time). When post hoc tests were pertinent, we used multiple comparisons with Bonferroni correction. a) $p<0,05$ by comparison to baseline values in the diet. b) $p<0,05$ between diets in the same time.
